# Human discrimination of head-centred visual–inertial yaw rotations

**DOI:** 10.1007/s00221-015-4426-2

**Published:** 2015-08-30

**Authors:** Alessandro Nesti, Karl A. Beykirch, Paolo Pretto, Heinrich H. Bülthoff

**Affiliations:** Department of Human Perception, Cognition and Action, Max Planck Institute for Biological Cybernetics, Tübingen, Germany; Research and Development, AMST Systemtechnik GmbH, Ranshofen, Austria; Department of Brain and Cognitive Engineering, Korea University, Seoul, Korea

**Keywords:** Differential thresholds, Multisensory integration, Vection, Self-motion perception, Yaw, Virtual reality, Psychophysics

## Abstract

To successfully perform daily activities such as maintaining posture or running, humans need to be sensitive to self-motion over a large range of motion intensities. Recent studies have shown that the human ability to discriminate self-motion in the presence of either inertial-only motion cues or visual-only motion cues is not constant but rather decreases with motion intensity. However, these results do not yet allow for a quantitative description of how self-motion is discriminated in the presence of combined visual and inertial cues, since little is known about visual–inertial perceptual integration and the resulting self-motion perception over a wide range of motion intensity. Here we investigate these two questions for head-centred yaw rotations (0.5 Hz) presented either in darkness or combined with visual cues (optical flow with limited lifetime dots). Participants discriminated a reference motion, repeated unchanged for every trial, from a comparison motion, iteratively adjusted in peak velocity so as to measure the participants’ differential threshold, i.e. the smallest perceivable change in stimulus intensity. A total of six participants were tested at four reference velocities (15, 30, 45 and 60 °/s). Results are combined for further analysis with previously published differential thresholds measured for visual-only yaw rotation cues using the same participants and procedure. Overall, differential thresholds increase with stimulus intensity following a trend described well by three power functions with exponents of 0.36, 0.62 and 0.49 for inertial, visual and visual–inertial stimuli, respectively. Despite the different exponents, differential thresholds do not depend on the type of sensory input significantly, suggesting that combining visual and inertial stimuli does not lead to improved discrimination performance over the investigated range of yaw rotations.

## Introduction

When moving through the environment, humans need to constantly estimate their own motion for performing a variety of crucial tasks (e.g. maintaining posture in presence of external disturbances or controlling a vehicle). This estimate of self-motion, computed by the central nervous system (CNS), is the result of complex multisensory information processing of mainly visual and inertial cues and is inevitably affected by noise, and therefore uncertainty. This, for example, can cause two motions with different amplitudes to be perceived as similar, or can cause repetitions of the same motion to be perceived as different.

Over the last century, researchers have been investigating the properties of this perceptual variability, as well as its sources. While a large group of important studies focused on measuring the smallest perceivable motion intensity (absolute threshold) and its dependency on motion direction and frequency (cf. Guedry 1974), only few studies addressed how the smallest perceivable *change* in motion intensity (differential threshold, DT) depends on the intensity of the supra-threshold motion (Zaichik et al. 1999; Mallery et al. 2010; Naseri and Grant 2012; Nesti et al. 2014a, 2015). DTs for different intensities of combined visual and inertial motion cues have (to the best of our knowledge) not been investigated yet, as previous studies focused on how visual and inertial sensory cues independently contribute to the discrimination of self-motion. In this study, we investigate the human ability to discriminate rotations centred on the head-vertical axis (yaw) by measuring DTs for different supra-threshold motion intensities in the presence of congruent visual–inertial cues. Moreover, by comparing DTs for visual–inertial rotation cues with DTs for visual-only and inertial-only rotation cues (measured as three separate conditions), we address the question of whether redundant information from different sensory systems can improve discrimination of self-motion.

### Supra-threshold motion discrimination

In everyday life, humans are frequently exposed to a wide range of self-motion intensities. For example during locomotion, head rotation velocities can range from 0 to 400 °/s and even higher (Grossman et al. 1988). Recent studies investigated human DTs for different motion intensities (Zaichik et al. 1999; Mallery et al. 2010; Naseri and Grant 2012; Nesti et al. 2014a, 2015). This is commonly done by presenting a participant with two consecutive motion stimuli and iteratively adjusting their difference in motion intensity until discrimination performance converges to a specific, statistically derived level of accuracy (Gescheider 1997). By measuring DTs for different reference intensities, these studies showed that DTs increase for increasing motion intensities.

In three recent studies, Mallery et al. (2010), Naseri and Grant (2012) and Nesti et al. (2014a) measured human DTs for inertial-only motion cues (i.e. in darkness) for head-centred yaw rotations, forward–backward translations and vertical translations, respectively. Moreover, Nesti et al. (2015) measured DTs for yaw self-motion perception as evoked by a purely visual stimulation (vection). These studies have shown that DTs can be described well by a power function of the general form Δ*S* = *k* * *S*^*a*^, where Δ*S* is the DT, *S* is the stimulus intensity and *k* and *a* are free parameters that depend on the type of motion investigated. Of these two parameters, the exponent is the one that determines how fast DTs change with intensity: an exponent of 0 reflects DTs that do not depend on stimulus intensity, whereas an exponent of 1 results in the well-known Weber’s law (Gescheider 1988), which linearly relates DTs to stimulus intensity. In the studies mentioned above, the exponent ranges from 0.37 for yaw discrimination (Mallery et al. 2010) to 0.60 for discrimination of upward translations (Nesti et al. 2014a). Whether the functions describing DTs for visual-only and inertial-only stimuli also hold for congruent visual–inertial stimuli is still open question that we address with the present work.

### Multisensory integration

In a natural setting, humans rely on visual, vestibular, auditory and somatosensory cues to estimate their orientation and self-motion. This information, coded by multiple sensory systems, must be integrated by the CNS to create a coherent and robust perception of self-motion. The theory of maximum likelihood integration (MLI) provides a mathematical framework for how noisy sensory estimates might combine in a statistically optimal fashion (Ernst and Bülthoff 2004; Doya et al. 2007). In addition to providing a prediction of the multisensory percept, MLI theory also predicts the variance (i.e. the uncertainty) associated with that percept, based on the individual variances associated with each sensory modality. According to MLI, multisensory estimates always have lower variances than individual unisensory estimates (Ernst and Bülthoff 2004; Doya et al. 2007).

MLI is supported by a large amount of experimental evidence, for example, in the fields of visual–auditory and visual–haptic integration (cf. Doya et al. 2007). However, it is not unusual for psychophysical studies on visual–inertial integration to report deviations, sometimes substantial, from MLI predictions. For example, De Winkel et al. (2010) measured the human ability to estimate heading from visual, inertial and congruent visual–inertial motion cues and observed that the variance associated with multimodal estimates was between the variances measured in the unisensory conditions. In a similar heading experiment, Butler et al. (2010) investigated human heading perception for visual and inertial stimuli as well as for congruent and incongruent visual–inertial stimuli. While congruent multisensory cues led to increased precision, for conflicting multisensory cues more weight was given to the inertial motion cue, resulting in multisensory estimates whose precision was not as high as MLI would predict. The MLI model was also rejected by De Winkel et al. (2013) in an experiment where participants discriminated between different yaw rotation intensities. In contrast, optimal or near-optimal integration of visual–inertial cues was reported in psychophysical experiments with humans (Butler et al. 2011; Prsa et al. 2012; Karmali et al. 2014), as well as monkeys (Gu et al. 2008; Fetsch et al. 2009). Interestingly, Butler et al. (2011) suggested that stereo vision might be important in order to achieve MLI of visual and inertial cues, although results from Fetsch et al. (2009) contradict this hypothesis.

Overall, considering the high degree of similarity between experimental setups and procedures, such qualitative differences in results are surprising. A possible explanation could reside in the intrinsic ambiguity of visual stimuli (De Winkel et al. 2010), which contain information on both object motion and self-motion. Depending on properties of the visual stimuli, such as their duration, participants may or may not experience illusory self-motion perception (vection) (Dichgans and Brandt 1978). If vection is absent or incomplete, sensory integration is not expected to occur since the two visual and inertial sensory channels are believed to inform about two different physical stimuli: the motion of objects in the visual scene and self-motion.

### Current study

The goal of this study is to psychophysically measure DTs for congruent visual–inertial yaw rotations over an intensity range of 15–60 °/s and to identify the parameters of an analytical relationship (power function) that relates yaw DTs to motion intensity. Furthermore, we measure, in a separate condition and with the same participants, DTs to yaw rotation in darkness. We then compare DTs measured for inertial motion cues (inertial-only condition) and visual–inertial motion cues (visual–inertial condition) with DTs measured for visual motion cues (visual-only condition). The latter data were collected in a previous experiment on vection during constant visual yaw rotations conducted in our laboratory (Nesti et al. 2015). The present study is therefore designed to facilitate comparison of the data with Nesti et al. (2015) and to allow testing of an MLI model that predicts the variance of the bimodal (visual–inertial) estimate based on the variance of the unimodal (visual-only and inertial-only) estimates. Note that the use of constant visual rotation is a drastic deviation from the standard approaches described above for investigating MLI of visual–inertial cues and is motivated by the desire to ensure that, in the presence of visual-only cues, participants’ discrimination is based on self-motion perception rather than object-motion perception. We hypothesize that DTs depend significantly on motion intensity and that providing visual–inertial motion results in DTs lower than those measured for unimodal motion cues, perhaps as low as MLI predicts.

This study extends current knowledge on self-motion perception by investigating motion discrimination with multisensory cues at different motion intensities. These types of stimuli occur frequently in everyday life and are therefore of interest to several applied fields. For instance, motion drive algorithms for motion simulators implement knowledge of self-motion perception to provide more realistic motion experiences within their limited workspace (Telban et al. 2005). Furthermore, models have been developed (Bos and Bles 2002; Newman et al. 2012) and employed to quantify pilots’ perceptions of self-motion and orientation during both simulated and real flight, allowing estimation of any perceived deviation from reality in the simulator. By measuring DTs, we provide necessary information to adapt these multisensory models to account for the effect of stimulus intensity on the perception of self-motion, which in turn will result in more accurate predictions particularly at high motion intensities.

## Methods

### Participants

Six participants (age 26–53, 1 female), four naïve and two experimenters (AN and KAB) took part in the study. They all had normal or corrected to normal vision, reported no history of balance or spinal disorders and no motion sickness susceptibility. Written informed consent was collected prior to the inclusion in the study, in accordance with the ethical standards specified by the 1964 Declaration of Helsinki.

### Setup

**Fig. 1:**
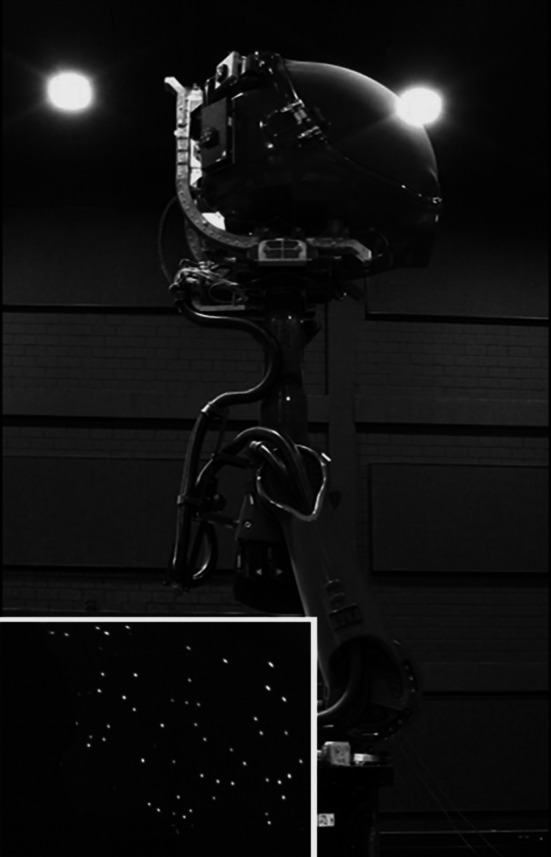
Experimental setup. Participants sat inside the simulator cabin and were presented with visual stimuli projected on the inner surface of the cabin door. The *inset* provides a picture of the visual stimulus

The experiment was conducted using the MPI CyberMotion Simulator, an 8 degrees-of-freedom motion system capable of reproducing continuous head-centred yaw rotations [Fig. 1, for technical details refer to Nieuwenhuizen and Bülthoff (2013); Robocoaster, KUKA Roboter GmbH, Germany]. Participants sat inside the closed cabin in a chair with a 5-point harness, and visual stimuli were presented on the white inner surface of the cabin door (approximately 60 cm in front of the participants’ head) by means of two chair-fixed projectors (each 1920 × 1200 pixels resolution, 60 Hz frame rate). For this experiment, a field of view of approximately 70° × 90° and an actual stimulus resolution of approximately 20 pixels/° were used. Participants wore headsets that played white noise during stimuli presentation to mask noise from the simulator motors and provide continuous communication with the experimenter (for safety reasons). The participant’s head was restrained with a Velcro band, which combined with careful instruction to maintain an upright posture helped participants avoid Coriolis effects (Guedry and Benson 1976; Lackner and Graybiel 1984), i.e. the illusory perception of rolling/pitching following head tilts during constant velocity yaw rotations. Participants controlled the experiment with a button box with three active buttons; one was used to initiate the stimulus (control button) and the other two for providing a forced-choice response (response buttons). As per instruction, the button box was held between the participants’ knees, an active effort to help minimize proprioceptive information from the legs. The seat was also wrapped in foam to help mask vibrations of the simulator.

### Stimuli

**Fig. 2:**
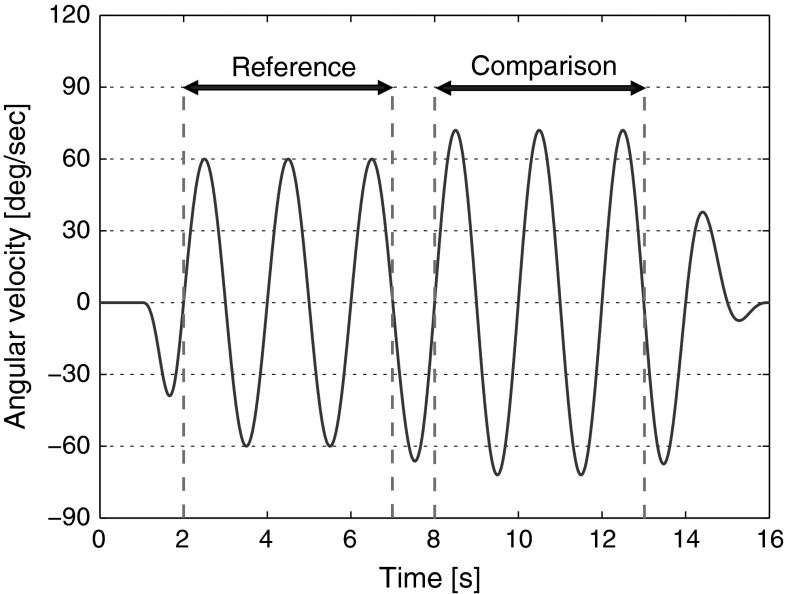
Velocity profile of a typical stimulus composed of a reference amplitude of 60 °/s and a comparison amplitude of 72 °/s

In both the visual–inertial and the inertial-only conditions, inertial stimuli consisted of 0.5 Hz sinusoidal yaw rotations centred on the participant’s head. Each stimulus was composed of two consecutive parts characterized by two different peak amplitudes, a reference amplitude and a comparison amplitude, whose presentation order was randomized. The stimulus velocity first increased from 0 °/s to the first peak amplitude following a raised half-cycle cosine mask of 1 s. This amplitude was then maintained for 5 s (2.5 cycles) before changing, again by means of a 1 s raised half-cycle cosine mask, to the comparison amplitude. After 5 s (2.5 cycles) the stimulus was terminated by decreasing its amplitude to 0 °/s through a 3 s raised half-cycle cosine mask. The velocity profile of a typical stimulus is illustrated in Fig. 2. Different stimulus onset and offset durations are used to hinder comparison of the two constant amplitudes based on stimulus accelerations. As shown by Mallery et al. (2010) through both modelling and experimental approaches, no confound is to be expected due to velocity storage for such stimuli, i.e. the perception of rotation that persists after the rotational stimulus stops (Bertolini et al. 2011). The stimuli designed for this study resemble those employed by Mallery et al. (2010) to the greatest possible extent to favour comparison of experimental findings.

Depending on the experimental condition, stimuli were always presented either in darkness (inertial condition) or combined with a virtual visual environment (visual–inertial condition) projected on the inner wall of the cabin (60 cm away from the participant). In the inertial condition, projectors were off and participants were instructed to close their eyes. Visual stimuli, generated with authoring software for interactive 3D applications (Virtools, 3DVIA), consisted of limited lifetime dots (Fig. 1) displayed on the surface of a virtual cylinder whose axis coincided with the head-vertical axis of the participants. The radius of the virtual cylinder (5 m) was chosen to achieve a satisfactory visual appearance on the screen (i.e. texture resolution and object size). Dot life was set to 1 s to ensure that no dot outlived a full cycle of the sinusoidal motion, thereby preventing participants from comparing dots’ travelled distances. The number of dots in the scene was maintained constant, and the appearance delay was selected randomly between 0 and 200 ms. Each dot’s diameter as it appeared on the inner wall of the cabin was 3 cm and remained constant for the entire lifetime of the dot. Visual and inertial sinusoidal rotations always had equal intensity and opposite direction, resulting in a congruent multisensory experience of self-motion, that is, the visual scene was perceived as earth-stationary. No visual fixation was used, thereby preserving the participants’ natural behaviour.

Similar to Nesti et al. (2015), participants were continuously rotating in each session around the head-vertical axis at the constant velocity of 20 °/s. Although the perception of constant inertial rotations disappears within a few seconds after rotation onset (Bertolini et al. 2011), such motion generates vibrations (vibration rms of 0.08 m/s^2^) unrelated to the stimulus, which serves multiple purposes. First, as suggested by Butler et al. (2010), when comparing reference and comparison stimuli, stimulus-unrelated vibrations could mask stimulus-related vibrations from the simulator, which are known to be amplitude dependent (Nesti et al. 2014b). Second, by setting the reference amplitude to 0 °/s, it is possible to measure the yaw absolute threshold in a discrimination task, as it prevents participants from merely performing a vibration detection task (Mallery et al. 2010; Merfeld 2011). Finally, this allows for a more direct comparison with DTs estimated by (Nesti et al. 2015). The direction of the constant rotation was reversed approximately every 15 min and stimulus presentation began 1 min after constant velocity was reached to guarantee disappearance of rotational motion perception.

An inertial measurement unit (YEI 3-Space Sensor, 500 Hz) mounted on top of a participant’s head was used to verify the absence of centripetal accelerations during constant and sinusoidal yaw rotations and for measuring temporal disparities between visual and inertial motion, a common concern for mechanical and visual systems. This procedure revealed that, when commanded simultaneously, the visual motion preceded the physical motion by approximately 32 ms. Because increasing temporal disparities diminishes the influence that multimodal cues have on each other (van Wassenhove et al. 2007; van Atteveldt et al. 2007), temporal disparities were minimized by delaying visual stimuli by 2 frames, which corresponds to approximately 33 ms at the projectors frame rate of 60 Hz.

### Procedure

Before stimulus presentation, participants sat in darkness (inertial condition) or in front of the visual environment, initially stationary with respect to the participants (visual–inertial condition). Stimuli were initiated by the participants through the button box and started 1 s after the control button was pressed. A 5 s tone accompanied the presentation of both the reference and the comparison amplitudes. After hearing a beep indicating the end of the stimulus, participants were asked “which rotation felt stronger (1st or 2nd)?”. Participants were specifically instructed to refer to the motion they felt during the two 5-s tone presentations and not during any other part of the stimulus. After a feedback beep, confirming that the answer was recorded, participants waited for 3 s before a beep signalled they could start the next stimulus. In the visual–inertial condition, the visual scene remained visible and stationary with respect to the participants during the time between stimuli.

**Fig. 3:**
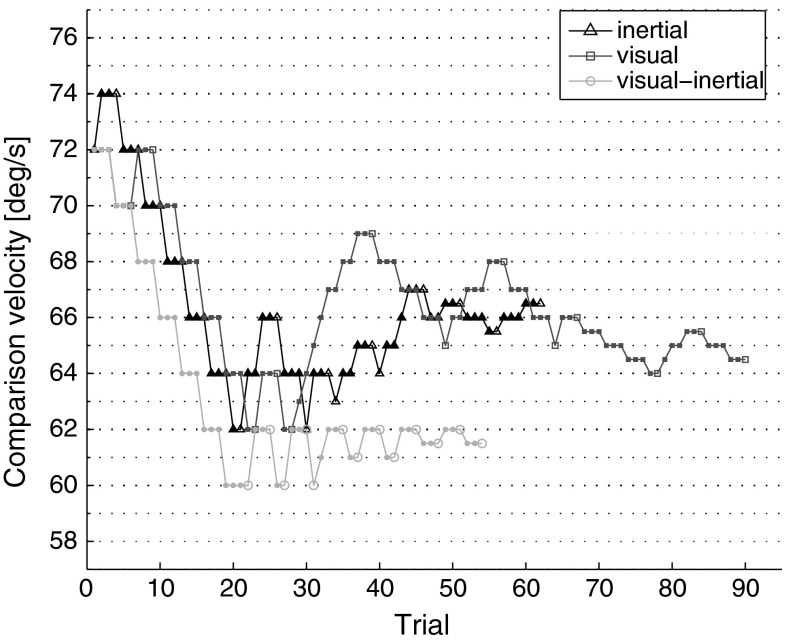
Evolution of the adaptive algorithms for one participant in the inertial (*black line*), and visual–inertial (*red line*) conditions. *Blue line* represents data re-plotted from Nesti et al. (2015), where DTs for visual-only motion cues were measured using an identical adaptive procedure. Reference velocity was 60 °/s. *Empty markers* indicate reversals (color figure online)

Both the inertial and the visual–inertial conditions were divided into four sessions of approximately 45 min each, with a 10 min break roughly in the middle of the session to avoid fatigue. Each participant was only allowed to complete 1 session per day. In every session, the participant’s DT was measured for one of the four reference velocities (15, 30, 45 or 60 °/s) using a psychophysical two-interval forced-choice (2IFC) procedure. While the reference velocity remained constant throughout the whole session, comparison velocities were adjusted for every trial according to an adaptive staircase algorithm: the stimulus level was decreased after three consecutive correct responses and increased after every incorrect response [3-down 1-up rule (Levitt 1971)]. Such an algorithm converges where the probability of a single correct answer is 0.794 (cube root of 0.5), i.e. when the probability of a stimulus increase (wrong answer) or decrease (three consecutive correct answers) is equal (*p* = 0.5). The comparison velocity *c*_0_ for the first trial was obtained by multiplying the reference velocity by 1.2. The step size, initially set at 2 °/s, was halved every five reversals. Sessions were terminated after 13 reversals (final step size of 0.5 °/s). Typical staircases for one participant are illustrated in Fig. 3. All participants completed the inertial-only condition before the visual–inertial condition. Reference velocities were tested in random order. An additional session was run to measure the yaw absolute threshold (reference velocity set to 0 °/s) for inertial-only motion stimuli. In this session, the initial comparison velocity was set to 2 °/s with a constant step size of 0.1 °/s.

### Visual-only condition

Human discrimination of yaw rotations in the presence of visual cues alone was investigated previously by Nesti et al. (2015), to allow comparison with inertial and visual–inertial cues, as was done in this work. Briefly, in Nesti et al. (2015) we measured DTs for circular vection for the same six participants of the present study and for the same four reference rotational velocities (15, 30, 45 and 60 °/s). The study also employed the same setup and experimental procedure (2IFC, 3-down 1-up adaptive staircase): at every trial participants experienced two consecutive stimuli and reported which rotation felt stronger. Visual rotations were presented at constant velocity, a stimulus that is known to induce a compelling self-motion perception due to its lack of conflict between visual and inertial information (Dichgans and Brandt 1978). Indeed, human perception of head-centred constant velocity inertial rotations in darkness decays to zero with time. After such time, this results in non-conflicting visual and inertial sensory information during constant visual rotations irrespective from the intensity of the inertial rotation. To guarantee that a compelling self-motion perception was induced in the participants *atevery trial*, visual rotations were terminated by participants via a button press only after the visual scene was confidently perceived as stationary, i.e. all the visual motion was attributed to self-motion. Note that this constitutes a qualitative difference with most published studies on MLI of visual–inertial cues in self-motion perception, where stimuli for the visual-only condition are obtained by simply removing the inertial component from the visual–inertial condition (Butler et al. 2010, 2011; De Winkel et al. 2010, 2013; Prsa et al. 2012). The method used in the present study to measure visual-only DTs has the benefit of avoiding a possible comparison of a true self-motion percept (for inertial-only and visual–inertial conditions) with a mixed perception of object and self-motion, which can occur with the other method. Although the stimuli from Nesti et al. (2015) differ from the stimuli employed in the present study in terms of stimulus frequency and visual environment, we argue that these differences do not hinder a meaningful comparison of the results of these two studies (see “Validity of study comparison” in “Discussion” section). A combined analysis allows for comparison of yaw discrimination in response to visual-only or inertial-only cues. Moreover, it allows for investigation of how redundant sensory information from the visual and inertial sensory systems combines in the presence of multisensory motion cues.

### Data analysis

For every condition, the last eight reversals of the staircase algorithm were averaged in order to compute the DT corresponding to the reference velocity and sensory modality tested. The DTs for each amplitude were averaged across participants for each of the three conditions, inertial-only, visual–inertial and visual-only (Nesti et al. 2015). The averages were fit for each condition to a power function of the form:

1 $$\Delta S = k*S^{a}$$

where Δ*S* is the differential threshold and *S* is the stimulus intensity and *k* and *a* are free parameters. The choice of the power function is motivated by previous studies showing that the power function provides a good description of DTs for self-motion perception as well as for other perceptual modalities (Guilford 1932; Mallery et al. 2010; Nesti et al. 2014a, 2015).

A repeated-measures analysis of the covariance (rmANCOVA) was run to assess the effect of the factor “condition” (3 levels: “inertial”, “visual” and “visual–inertial”) and of the covariate “motion intensity”. In order to perform the rmANCOVA using the power function model, the following transformation of the data was required:

2 $$\log \left( {\Delta S} \right) = \log \left( k \right) + a*\log \left( S \right)$$

Additionally, to assess whether the integration of visual and inertial cues followed the MLI model in this experiment, an rmANCOVA was run to compare participants’ DTs in the visual–inertial condition with MLI predictions based on their own DTs as measured in the visual-only and inertial-only conditions. Note that it is common practice to test MLI using the variance of the physiological noise underlying the decision process rather than the experimentally derived thresholds (see, e.g. Butler et al. 2010; De Winkel et al. 2013). For a two-interval discrimination task, such as the one employed here, this requires dividing the DTs by 0.58 (Merfeld 2011). Such a linear transformation of the data does not, however, affect the results of the statistical analysis, and we therefore test MLI directly on the measured DTs using the following equation (Ernst and Bülthoff 2004):

3 $$\overline{{{\text{DT}}_{\text{vi}} }}^{2} = \frac{{{\text{DT}}_{\text{v}}^{2} * {\text{DT}}_{\text{i}}^{2} }}{{{\text{DT}}_{\text{v}}^{2} + {\text{DT}}_{\text{i}}^{2} }}$$

where ${\text{DT}}_{\text{v}}$ and ${\text{DT}}_{\text{i}}$ are the DTs measured in the visual-only and inertial-only conditions, respectively, for every reference intensity and $\overline{{{\text{DT}}_{\text{vi}} }}$ is the MLI prediction for the DT at the given reference velocity in the visual–inertial condition.

### Stimulus noise analysis

When reproducing motion commands, motion simulators inevitably introduce noise that affects the amplitude and spectral content of the intended inertial stimulus and could affect psychophysical measurements (Seidman 2008; Chaudhuri et al. 2013). As extensively discussed in Nesti et al. (2014b), analysing the noise introduced in the stimulus by the simulator provides important insights into the study of self-motion perception, as it allows dissociation of the mechanical noise of the experimental setup from the noise that is inherent in the perceptual processes. A signal-to-noise ratio (SNR) analysis (Nesti et al. 2014b) of the motion stimuli was therefore conducted using an inertial measurement unit (STIM300 IMU, Sensonar AS, 250 Hz) rigidly mounted on the floor of the simulator cabin. The SNR expresses the relative amount of commanded signal with respect to motion noise and is therefore an indicator of similarity between commanded and reproduced motion. For every reference velocity, 20 stimulus repetitions were recorded and the noise was then extracted by removing the motion command from the recorded signal (Nesti et al. 2014b). Average SNRs were computed for every reference stimulus and tested by means of an ANCOVA to investigate the effect of motion intensity on the motion SNRs.

4 $${\text{SNR}} = \left( {\frac{{{\text{rms}}_{\text{signal}} }}{{{\text{rms}}_{\text{noise}} }}} \right)^{2}$$

where rms stands for the root mean square of the noise signal and of the recorded signal (Nesti et al. 2014b).

## Results

**Fig. 4:**
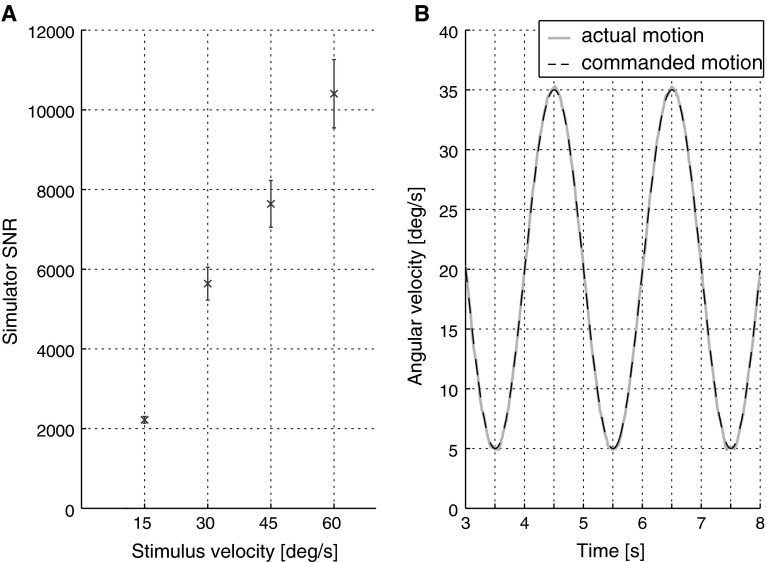
SNR analysis of the motion stimuli employed in this study. **a** SNRs increase for increasing rotational intensities, resulting in the highest comparison stimulus (peak amplitude of 60 °/s) having an SNR approximately five times higher than the lowest comparison stimulus (peak amplitude of 15 °/s). *Error bars* represent ±1 SEM. **b** Comparison of the commanded and recorded motion profile for one stimulus with 15 °/s amplitude

Motion analysis of the reference stimuli, illustrated in Fig. 4, shows a significant increase in stimulus SNR for increasing amplitudes of the velocity command (*F*(1,78) = 113.8, *p* < 0.001). This is a common feature of motion simulators (cf. Nesti et al. 2014b) and is expected to facilitate motion discrimination of higher as compared to lower motion intensities for those perceptual systems [including the human perceptual system (Greig 1988)] whose discrimination performance increases with SNRs. The fact that human DTs for self-motion increase for increasing motion intensities (Mallery et al. 2010; Naseri and Grant 2012; Nesti et al. 2014a, present study) could indicate an additional noise source inherent to the perceptual system and proportional to stimulus intensity.

During the experiment, each condition took approximately 40 min and required 61 trials on average. No session needed to be terminated because of fatigue or other reasons, and no participant reported symptoms of motion sickness.

The absolute threshold measured in the inertial-only condition was 0.87 ± 0.13 °/s, a value that is consistent with previous studies (see, e.g. Zaichik et al. 1999; Mallery et al. 2010; Valko et al. 2012; Roditi and Crane 2012).

**Fig. 5:**
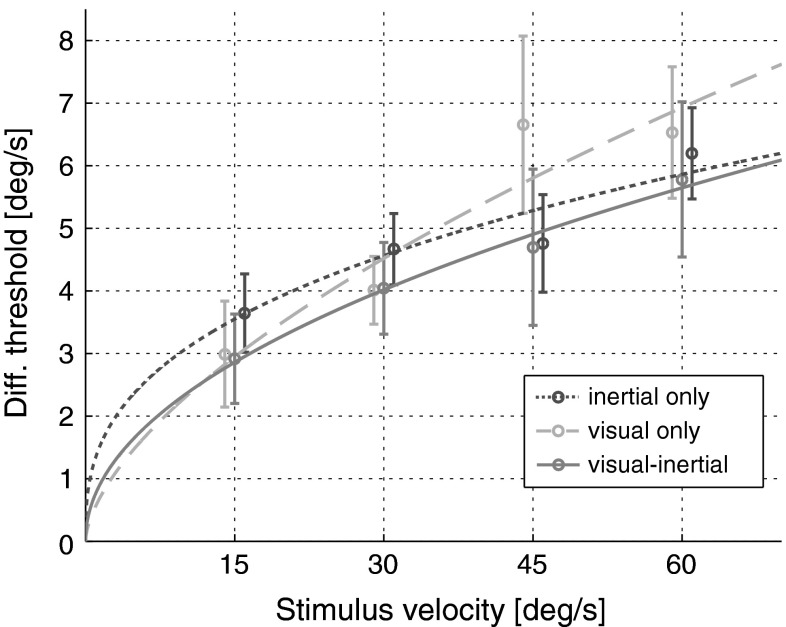
DTs for yaw rotations with inertial (*blue*), visual (*green*) and visual–inertial (*red*) motion cues are well described by three power functions. DTs for visual cues are re-plotted from Nesti et al. (2015). *Error bars* represent ±1 SEM (color figure online)

Fitting Eq. 1 (power function) to inertial, visual and visual–inertial DTs averaged for each reference velocity results in gain coefficients *k*_i_, *k*_v_ and *k*_vi_ of 1.33, 0.55 and 0.76 and in exponent coefficients *a*_i_, *a*_v_ and *a*_vi_ of 0.36, 0.62 and 0.49, where the subscripts i, v and vi stand for inertial, visual and visual–inertial, respectively (Fig. 5). Goodness of fit is quantified by *R*^2^ coefficients of 0.88, 0.89 and 0.99, respectively. Note that the inertial condition qualitatively replicates the findings of Mallery et al. (2010), where *k*_i_ = 0.88 and *a*_i_ = 0.37. The overall higher thresholds found in our study, reflected in the higher gain (1.33 vs 0.88), are likely due to the use of a different simulator. However, similar exponents indicate that the effect of motion intensity on self-motion discrimination in darkness is consistent between studies. This is only partially surprising given the high level of similarity in the experimental methods. A linear fit resulted in intercept coefficients *q*_i_, *q*_v_ and *q*_vi_ of 2.88, 1.73 and 2.05 and in slope coefficients *m*_i_, *m*_v_ and *m*_vi_ of 0.05, 0.09 and 0.06. *R*^2^ coefficients are 0.91, 0.87 and 0.99 in the inertial-only, visual-only and visual–inertial condition, respectively. Although the linear model provides a slightly superior fit than the power function model for the inertial-only condition, we performed the rmANCOVA using the power function model as it should generalize better for larger ranges of sensory input amplitudes (Guilford 1932; Teghtsoonian 1971).

**Fig. 6:**
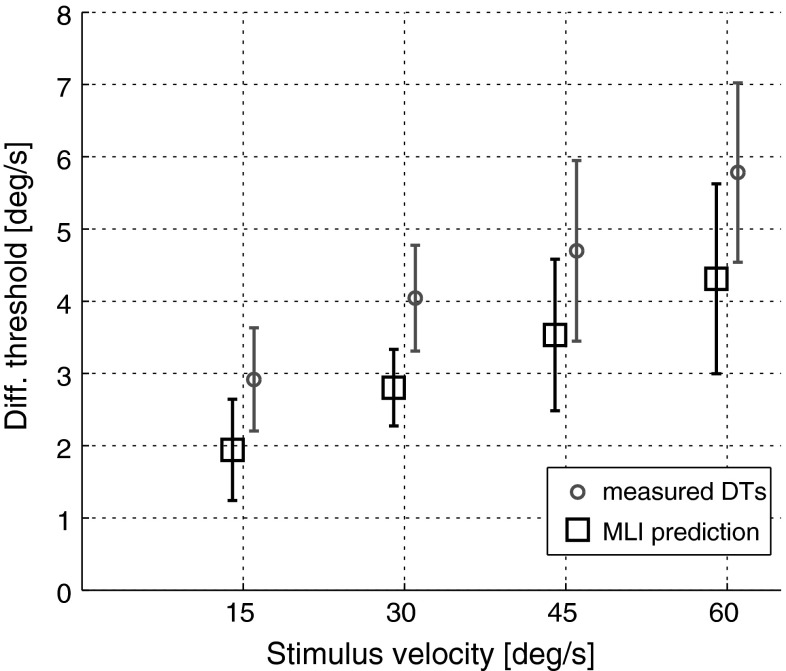
Comparison of measured DTs (*red circles*) and predicted DTs (*black squares*) based on MLI. Data do not support models of statistically optimal integration of visual and inertial sensory information. *Error bars* represent ±1 SEM (color figure online)

The ANCOVA revealed that DTs increased significantly with motion intensity (*F*(1,63) = 32.55, *p* < 0.001), confirming previous results on self-motion discrimination in the presence of visual-only or inertial-only cues and extending the analysis to the case of visual–inertial cues. However, DTs did not depend on the cue type (*F*(2,63) = 1.59, *p* = 0.21), i.e. whether participants experienced inertial, visual or visual–inertial stimuli. Predictions based on MLI were contradicted by measured visual–inertial DTs (Fig. 6), with actual results significantly higher (*F*(1,40) = 5.93, *p* = 0.02).

## Discussion

Human self-motion perception involves the contribution of different sensory information from the visual, vestibular, auditory and somatosensory systems. In this study, we investigated human discrimination of self-motion for a wide intensity range of yaw rotations in darkness (inertial-only motion cues) and with congruent visual–inertial motion cues. Measured DTs increase with motion intensity following a trend described well by a power function, in agreement with previous studies on rotations and translations in darkness (Mallery et al. 2010; Naseri and Grant 2012; Nesti et al. 2014a) and for visually induced self-motion perception (Nesti et al. 2015). The use of a power function is consistent with previous work on self-motion perception (Mallery et al. 2010; Nesti et al. 2014a, 2015) and resulted in a high goodness of fit. Note, however, that a Weber’s law fit also provides a similar goodness of fit.

In the next sections, the relationship between DTs and motion intensity and the sub-optimal integration that emerged from the present study are discussed in detail.

### Discrimination of yaw rotations

Constant discrimination performance (i.e. constant DTs) would be expected if the relationship between physical and perceived motion intensity was linear and affected by constant noise. Instead, we found that human DTs for self-motion are not independent from the intensity of the motion but rather increase for increasing motion intensities. The present study shows that such behaviour is present not only for visual-only (Nesti et al. 2015) and inertial-only conditions (Zaichik et al. 1999; Mallery et al. 2010; Naseri and Grant 2011; Nesti et al. 2014a, present study), but is encountered also for congruent visual and inertial sensory cues. This indicates that the perceptual processes converting physical to perceived motion are nonlinear and/or affected by stimulus-dependent noise (with the amount of noise increasing with the intensity of the physical stimulus). In contrast, responses to head rotations are linear with constant inter-trial variability for neurons in the vestibular afferents (Sadeghi et al. 2007), as well as for eye movements (Pulaski et al. 1981; Weber et al. 2008). A comparison between psychophysical and physiological studies suggests therefore that nonlinearities and/or stimulus-dependent increases in physiological noise occur further along the neuronal pathways processing sensory information and are likely due to central processes, multisensory integration mechanisms and/or cognitive factors. Interestingly, increased variability was observed in neural recordings from the vestibular nuclei of macaque monkeys for faster compared to slower inertial (Massot et al. 2011), visual (Waespe and Henn 1977) and visual–inertial (Allum et al. 1976) yaw rotational cues. We hypothesize that this increase in variability reduces discrimination performance at high stimulus velocities. Future studies are required to better quantify the relationship between stimulus intensity, neural activity and behavioural responses.

**Fig. 7:**
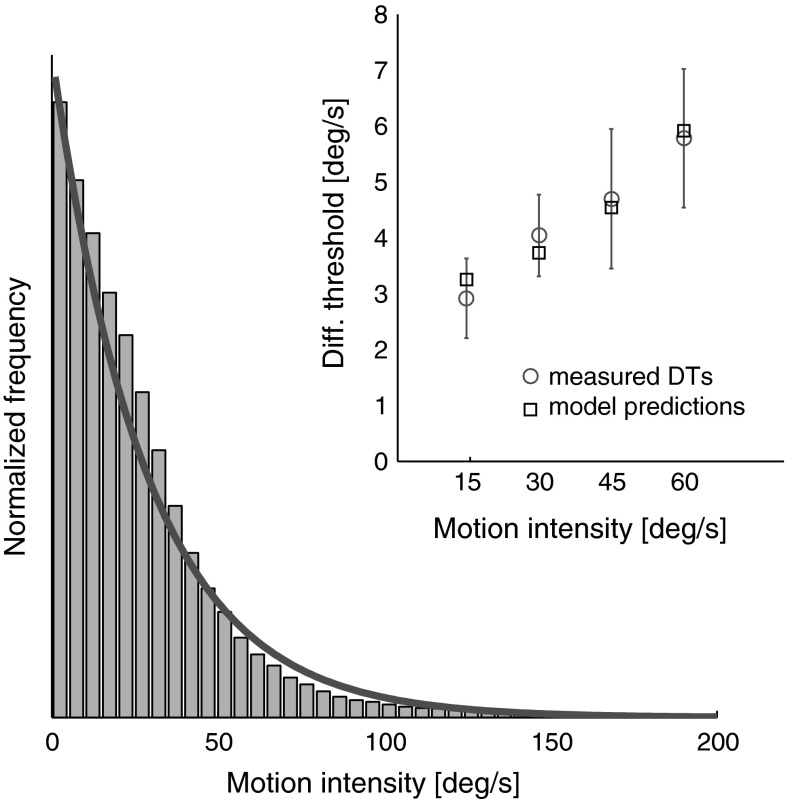
Physical stimulus statistics obtained using an IMU are presented in a *histogram* where *bars* represent the normalized occurrence frequency of yaw rotational velocities during a 40 min running session. Normalized frequencies are obtained by dividing the *histogram* of yaw data samples by its area. Fitting data with an exponential distribution [*red line*, *y*(*S*) = 28.5 * exp(−28.5 * *S*), where *S* is the stimulus intensity and *y*(*S*) is the exponential distribution] allows development of a simple model [Δ*S* = *a* + *b* * 1/*y*(*S*)] that relates DTs to motion intensity by accounting for how frequently a particular intensity occurs. *Error bars* represent ±1 SEM (color figure online)

Stimulus-dependent DTs might also represent an efficient strategy of the CNS to account for how frequently a particular motion intensity occurs in everyday life. This would indeed result in smaller DTs for low rotation intensities, as they are more common than large rotations during everyday experience. To better illustrate this concept, we present in Fig. 7 rotational velocity intensities recorded with an inertial sensor (YEI 3-Space Sensor, 500 Hz) over 40 min of normal activity (running) and fit with an exponential distribution. A simple model with two parameters, gain and offset, is able to describe the increasing trend of DTs well.

Note that the simple model from Fig. 7 only serves as an illustrative example. A more systematic approach for using stimulus statistics to model perceptual responses is presented in Wei and Stocker (2013).

### Multisensory integration

In this study, we investigated multisensory integration in a yaw intensity discrimination task by comparing DTs for inertial-only and visual-only motion stimuli with DTs for congruent (i.e. redundant) visual–inertial cues. Although a number of studies indicated MLI as a valid model of visual–inertial cue integration for the perception of translational and rotational motion (see, e.g. Gu et al. 2008; Fetsch et al. 2009; Butler et al. 2011; Prsa et al. 2012; Karmali et al. 2014), our data do not seem to follow MLI. This is only partially surprising, as we are not the first to report substantial deviations from MLI (Telford et al. 1995; Butler et al. 2010; De Winkel et al. 2010, 2013). However, when comparing this study with the existing literature, it is important to consider two main differences. First, the great majority of visual–inertial integration studies used a heading task, rather than a rotation intensity discrimination task as we have. Although MLI has been suggested as a general strategy for multisensory integration, the stimuli are radically different and even involve different vestibular sensors (note that a heading stimulus is composed by linear translations only); therefore, caution is advised in the generalization of the results. The only other studies, of which we are aware, that employed yaw stimuli are from Prsa et al. (2012), whose findings support MLI, and from De Winkel et al. (2013), where the MLI model is rejected. Second, the stimuli we chose for testing for MLI were designed to avoid visual–inertial conflicts. This required an inertial-only stimulus to which the visual system is insensitive (i.e. motion in darkness) and a visual-only stimulus to which the inertial systems are insensitive (i.e. rotation at constant velocity (Nesti et al. 2015), which lacks inertial accelerations). To the best of our knowledge, such stimuli have not been previously employed for validating MLI of visual–inertial motion cues. Instead, perceptual thresholds for visual-only cues were always investigated by removing the inertial component from the visual–inertial stimulus, a choice that has the clear benefit of minimizing experimental manipulations but might lead to visual–inertial sensory conflicts.

In the light of our experimental results, the visual–inertial DTs may be reconciled with MLI through the theory of causal inference (Beierholm et al. 2008; Shams and Beierholm 2010), which predicts that sensory integration is subordinate to whether stimuli are perceived as originating from the same physical event or not. Although in the present study, the visual and inertial stimuli were always congruent in representing head-centred rotations, we have to consider the possibility that they were not always perceived as congruent by the participants. Indeed, the simple fact that visual stimuli were computer-generated virtual objects might induce in the participants expectations of incongruence with the actual motion (the visual and inertial stimuli “belong” to different environments). Causal inference theory suggests that in this event, stimuli are segregated and participants respond based on the information coming from either one of the two sensory channels. Statistical models, other than causal inference, have been suggested in the literature to account for the possibility that stimuli are not integrated according to MLI because they are perceived as incongruent [see De Winkel et al. (2013) for a review]. For instance, a “switching strategy” model could be applied to our data by assuming that stimuli perceived as congruent are integrated according to MLI, whereas stimuli perceived as incongruent are segregated and the response is based only on one sensory modality (e.g. the inertial). Equation 3 would then be modified as follows:

5 $$\overline{{\sigma_{\text{iv}} }}^{2} = \frac{{\sigma_{\text{i}}^{2} * \sigma_{\text{v}}^{2} }}{{\sigma_{\text{i}}^{2} + \sigma_{\text{v}}^{2} }}* \pi + \sigma_{\text{i}}^{2} *\left( {1 - \pi } \right)$$

leading to an estimated average probability (*π*) of 0.33 that participants perceived the stimuli as congruent.

### Nonlinear self-motion perception models

Human self-motion perception models compute how people update the estimate of their motion in space in response to physical motion. Several models were developed combining knowledge of sensor dynamics, oculomotor responses, psychophysics and neurophysiology (Merfeld et al. 1993; Bos and Bles 2002; Zupan et al. 2002; Newman et al. 2012). Despite capturing a large variety of perceptual phenomena well, to the best of our knowledge no published model can account for the decrease in discrimination performance with increasing motion intensity. The experimental data collected in this study and by Nesti et al. (2015) constitute a crucial step towards a more complete approach to self-motion perception models. Considering that the DTs measured here increase with stimulus intensity and were not affected by manipulation of the type of sensory information, a natural and straightforward choice would be to implement a single, common nonlinear process after the integration of the visual and inertial sensory pathways. Future studies should be dedicated to measuring rotational and translational multisensory DTs for the remaining degrees of freedom, implementing perceptual nonlinearities in computational models of human self-motion perception and validating these models using alternative motion profiles and experimental paradigms (e.g. maximum likelihood difference scaling, Maloney and Yang 2003).

### Validity of study comparison

We compared our results with DTs for vection (Nesti et al. 2015) to test the hypothesis that redundant information from the visual and inertial sensory systems is perceptually combined in a statistically optimal fashion. Comparison of DTs measured here and in Nesti et al. (2015) is particularly natural because of the high similarity between the studies (experimental setup, participants, procedure and stimulus intensities). However, two important differences should be discussed.

First, in the present study 0.5 Hz sinusoidal motion profiles were used, whereas in Nesti et al. (2015) we measured vection DTs for constant (0 Hz) yaw rotations and stimuli were self-terminated by the participant to account for the high individual variability in vection onset time (Dichgans and Brandt 1978). These choices were made in order to measure DTs for stimuli as free of visual–inertial conflicts as possible, ensuring that all the visual motion is attributed to self-motion rather than object motion. Note how, for supra-threshold motion intensities, a visual stimulus at 0.5 Hz combined with no inertial motion will surely evoke a visual–inertial sensory conflict, as the continuous changes in the velocity of the visual environment conflict with the lack of acceleration signal from the inertial sensory systems. Evidence that conflicts between visual and inertial cues could confound self-motion perception is provided for instance by Johnson et al. (1999), who showed that in bilateral labyrinthectomized patients, who lack one of the main sources of inertial information (i.e. the vestibular system), vection latencies are shorter than those of healthy subjects. Comparing DTs for constant rotations with DTs for visual–inertial rotations at 0.5 Hz requires, however, the assumption that visual responses remain constants within this frequency range. Previous studies indicate that postural, psychophysical and neurophysiological responses to visually simulated self-motion show low-pass characteristics (Robinson 1977; Mergner and Becker 1990; Duh et al. 2004). For instance, visual responses in the vestibular nuclei only begin to attenuate for frequencies higher than 0.03 Hz (Robinson 1977), while subjective reports of circular vection intensities remain approximately constant for frequencies between 0.025 and 0.8 Hz (Mergner and Becker 1990). It is, however, reasonable to expect that this attenuation is at least in part due to multisensory conflicts that arise at stimulus frequencies to which the inertial sensors respond. Further studies in labyrinthectomized patients might help in clarifying the dependency of visual responses on frequency, although it should not be forgotten that the vestibular system is not the only system contributing to self-motion perception.

The second important difference involves the different visual stimulus: whereas in the present study we employed a limited lifetime dot field, in Nesti et al. (2015) we employed a 360° panoramic picture of a forest. Although it is known that different visual environments (e.g. with different spatial frequencies) affect vection onset time (Dichgans and Brandt 1978), we suggest that DTs after vection arises (i.e. when the visual environment is perceived as stationary) depend only on the velocity of the optic flow and not on the texture of the visual stimulus. This difference could be obviously eliminated in future studies by employing the same virtual environment for every condition and ensuring that it does not provide visual references.

To the best of our knowledge, this is the first study that focuses on minimizing sensory conflicts when testing MLI of visual–inertial cues for self-motion perception. While we acknowledge that the discussed differences between stimuli in the three conditions advise for caution in the interpretation of the results, we believe that preventing confounds between object-motion and self-motion perception in psychophysical experiments is an important step towards the understanding of the perceptual processes underlying the integration of visual–inertial cues.
